# A case report and literature review of duodenal adenocarcinoma with complete loss of mismatch repair proteins

**DOI:** 10.3389/fonc.2026.1749558

**Published:** 2026-02-19

**Authors:** Jie Ao, Xiangfeng Zhu, Yuan Zhao, Mimi Zhao, Yi Luo, Qiushi Wang

**Affiliations:** 1 Department of Pathology, Daping Hospital, Army Medical University, Chongqing, China; 2 Department of Radiology, Daping Hospital, Army Medical University, Chongqing, China; 3 Department of Nuclear Medicine, Daping Hospital, Army Medical University, Chongqing, China

**Keywords:** duodenal adenocarcinoma, high microsatellite instability, mismatch repair deficiency, somatic mutation, tumor mutational burden

## Abstract

**Background:**

Deficient mismatch repair (dMMR)/microsatellite instability-high (MSI-H) phenotype holds significant prognostic and therapeutic implications in gastrointestinal cancers. While typically characterized by loss of specific MMR protein pairs, the concomitant loss of all four MMR proteins (MLH1, PMS2, MSH2, MSH6) is scarcely documented, with molecularly confirmed cases being exceptionally rare.

**Case presentation:**

We presented a case of duodenal adenocarcinoma whose contrast-enhanced 64-row CT and PET/CT demonstrated characteristic features highly suspicious for malignancy. Subsequently, the patient underwent duodenal resection. Histopathology showed moderately differentiated adenocarcinoma invading the subserosa and immunohistochemical staining revealed MLH1, PMS2, MSH2 and MSH6 were completely lost in tumor cells. Molecular profiling confirmed MSI-H status, high tumor mutational burden (TMB-H), MLH1 promoter hypermethylation and somatic mutations in MSH2 and MSH6 genes, but without pathogenic germline variants.

**Conclusion:**

We represent a molecularly validated case of dMMR duodenal adenocarcinoma, suggesting a somatic molecular pathogenesis distinct from classic intestinal cancer. The findings highlight the critical importance of comprehensive molecular characterization in rare tumors, as accurate identification of these phenotypes are important for optimal treatment selection, particularly immune checkpoint inhibitor therapies for gastrointestinal malignancies.

## Introduction

Duodenal adenocarcinoma is a rare malignancy of the small intestine, whose molecular pathogenesis is less elucidated compared to colorectal cancer (CRC) (1). A pivotal role in the molecular pathogenesis of intestinal cancers is played by defects in the DNA mismatch repair (MMR) system. The core of this system consists of protein complexes formed by MLH1, PMS2, MSH2, and MSH6, which function to identify and rectify errors occurring during DNA replication, thereby maintaining the stability of genomic microsatellite sequences (2, 3). Deficiency of MMR system (dMMR) leads to high microsatellite instability (MSI-H), which in turn drives tumorigenesis (2–4).

A strong agreement exists between MSI and MMR status (5). dMMR or MSI-H status carries significant clinical implications in CRC: such tumors often exhibit distinct pathological features, a specific clinical course, and demonstrate remarkable sensitivity to immune checkpoint inhibitors, predicting favorable treatment responses and prognosis (6).

It is noteworthy that the vast majority of intestinal cancer patients identified as dMMR show only a partial loss of protein expression (7, 8). The most common patterns are concurrent loss of MLH1 and PMS2 or concurrent loss of MSH2 and MSH6 (9). Isolated loss of a single protein is also observed. MLH1 promoter hypermethylation, while uncommon in the overall landscape of small bowel adenocarcinomas(SBAs), is a defining and important event in the majority of sporadic dMMR cases (10). However, the concurrent and complete loss of all four MMR proteins is extremely rare. Singh Aminder et al. identified 2 cases of colorectal cancer with pan-MMR loss, and compared the consistency between MMR and MSI, but the mechanism underlying the loss of all four MMR proteins has not been elucidated (11). Satoko Kageyama et al. reported a case of SBA with complete loss of all four MMR proteins, whose genetic testing revealed Lynch syndrome (LS) with a germline pathogenic variant in MSH2 (12). However, there’s no comprehensive molecular profile reported in duodenal adenocarcinoma with pan-MMR loss. A recent review emphasizes that molecular subtyping of gastrointestinal tumors is fundamental to precision medicine, enabling a shift from one-size-fits-all treatments to biomarker-driven strategies like immune checkpoint inhibitors for MSI-H/dMMR profiles and targeted therapies for specific mutations, which significantly improves outcomes for molecularly defined patient subsets (13).

Here we report a rare case of duodenal adenocarcinoma pathologically confirmed to lack all four MMR proteins in tumor cells. By detailing this case’s clinical presentation, pathological features, molecular alterations, and reviewing the relevant literature, we hope to deepen the understanding of this peculiar molecular phenotype of complete MMR protein loss, explore its potential pathogenic mechanisms.

## Case presentation

### Clinical history and imaging findings

**Figure 1:**
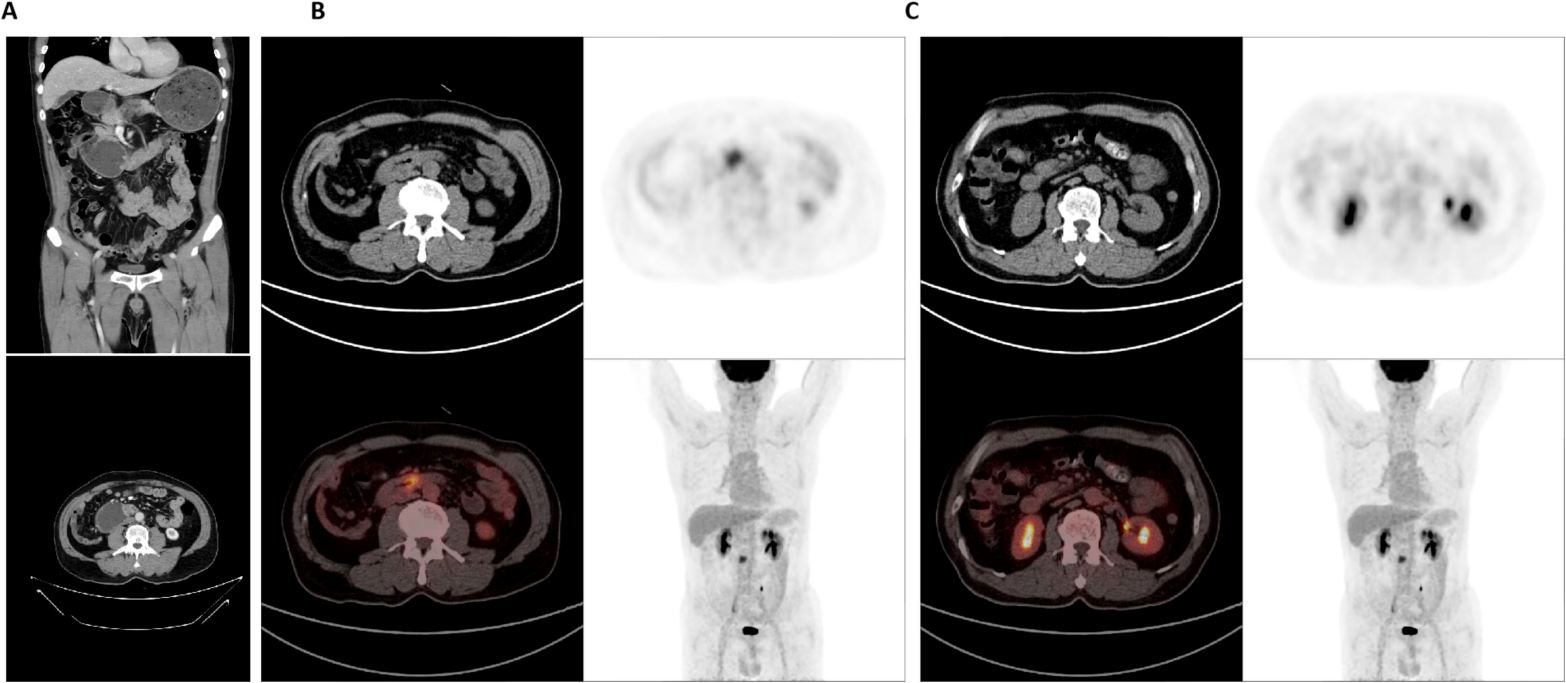
Contrast-enhanced abdominal CT and PET/CT imaging of the duodenal mass. **(A)** CT image demonstrates circumferential wall thickening of the horizontal segment of the duodenum, showing heterogeneous enhancement; and reveals proximal duodenal and gastric dilation with fluid and content retention, indicative of high-grade obstruction. **(B)** PET/CT image highlights the hypermetabolic duodenal lesion and significantly increased FDG uptake within the thickened duodenal wall, highly suggestive of malignancy. **(C)** PET/CT shows no abnormal metabolic activity was observed in regional lymph nodes or distant metastases. CT, chest enhanced computed tomography; PET, positron-emission tomography.

A 57-year-old male patient presented with a one-month history of persistent abdominal distension, acid reflux, and vomiting. Physical examination revealed mild tenderness in the epigastric region without a palpable mass. Contrast-enhanced 64-row CT demonstrated localized wall thickening in the third portion (horizontal segment) of the duodenum, accompanied by luminal narrowing and heterogeneous enhancement during both arterial and portal venous phases (**Figure 1A**). Significant proximal duodenal dilation with fluid retention and gastric content retention was observed, suggesting high-grade intestinal obstruction. The imaging findings confirmed full-thickness invasion of the duodenal wall and involvement of the periduodenal fat plane. No evidence of distant metastasis to the liver or peritoneum was identified at the time of initial diagnosis.

PET/CT revealed abnormal bowel wall changes in the horizontal duodenum with significantly increased FDG uptake, which was highly consistent with malignant tumor characteristics based on clinical context (**Figure 1B**). Several small periduodenal lymph nodes were noted without increased FDG avidity (**Figure 1C**), suggesting reactive hyperplasia rather than metastatic involvement.

The clinical and imaging features were highly suggestive of malignant duodenal neoplasia. Subsequent histological examination of the biopsy specimens confirmed the diagnosis of adenocarcinoma. Following diagnosis, the patient underwent duodenal resection.

### Histopathological diagnosis​

**Figure 2:**
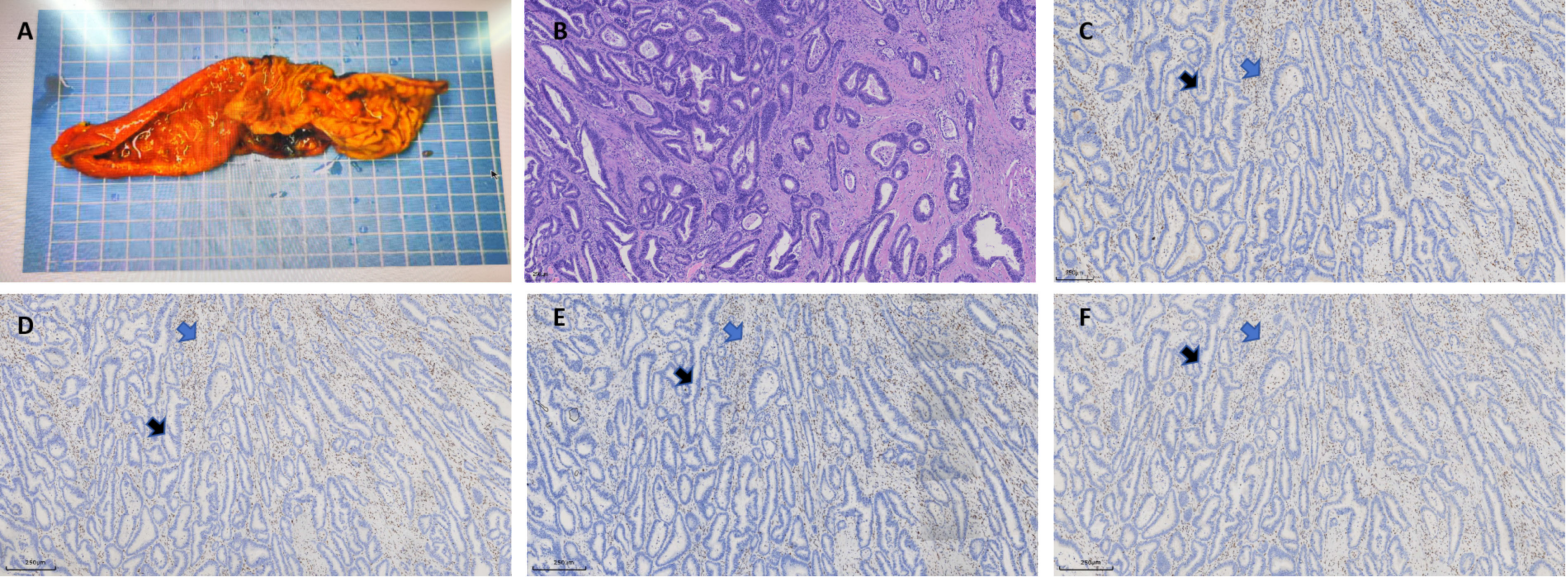
Histopathological and immunohistochemical **(IHC)** findings of the duodenal adenocarcinoma. **(A)** Gross specimen of the resected duodenum and tumor. **(B)** Hematoxylin and eosin (H&E) staining reveals moderately to poorly differentiated adenocarcinoma with irregular glandular structures and stromal desmoplasia (original magnification ×250). **(C-F)** IHC staining for MMR proteins performed on consecutive sections of the tumor sample and results demonstrates complete loss of nuclear expression in tumor cells (blue arrows) for MLH1 **(C)**, MSH2 **(D)**, MSH6 **(E)** and PMS2 **(F)**, with intact staining in internal controls (black arrows) (original magnification ×250).

The submitted duodenal segment measured approximately 15.3cm in length and 3.2cm in maximum diameter. An ulcerated mass was identified 2.9cm from the proximal margin and 10.5cm from the distal margin, measuring 2.2×1.8×1.1cm (**Figure 2A**). The mass involved the full thickness of the intestinal wall. Histopathological examination revealed a tumor (pT3N0Mx) composed of irregular glandular/tubular structures with areas showing fused and cribriform glandular patterns (**Figure 2B**). Focal solid/sheet-like growth patterns were observed. Tumor cells exhibited round to oval nuclei with hyperchromasia, discernible nucleoli, and frequent mitotic figures. No definite lymphovascular invasion or perineural invasion was identified.

Immunohistochemical staining for the MMR proteins revealed that MLH1, PMS2, MSH2 and MSH6 were completely lost in tumor cells, with preserved nuclear staining in internal control cells (**Figures 2C–F**), which confirmed the technical adequacy and reliability of the assay. These results indicated a dMMR status.

### Molecular diagnosis

**Figure 3:**
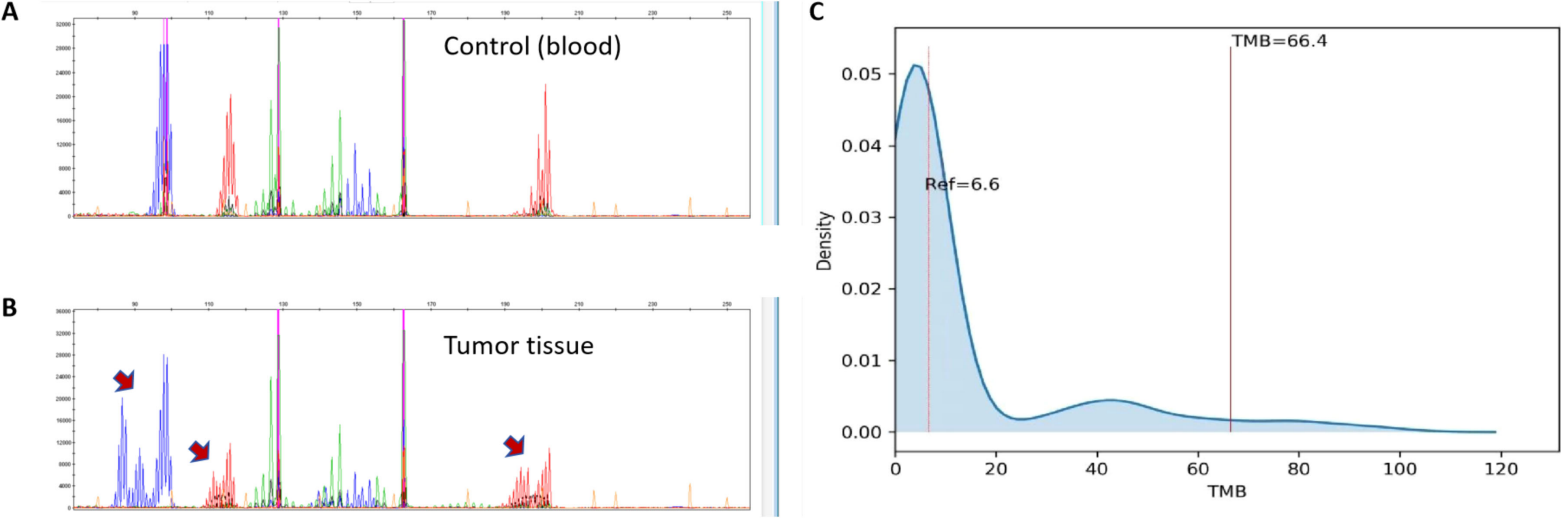
Molecular results of the duodenal adenocarcinoma. **(A, B)** Capillary electrophoresis chromatogram from microsatellite instability (MSI) testing by PCR. The tumor sample **(B)** demonstrates shifts in electrophoretic mobility compared to the matched blood control **(A)** at mononucleotide sites BAT-25 and BAT-26, and a dinucleotide D2S123 (red arrows), confirming a high level of microsatellite instability (MSI-H). **(C)** Next-generation sequencing (NGS) data demonstrates a high tumor mutational burden (TMB-H).

Molecular analysis further confirmed the dMMR status of the tumor. Polymerase chain reaction (PCR) and capillary electrophoresis test showed MSI-H status (**Figures 3A, B**). High-throughput next-generation sequencing (NGS) was performed for both tumor tissues and normal tissues, which was conducted on the Illumina high-throughput sequencing platform to analyze 556 cancer hotspot genes, using the UCSC hg19 (February 2009) human reference genome for alignment. The results identified pathogenic somatic mutations in MSH2 (p.A328fs) and MSH6 (p.T1085fs) (**Table 1**). No pathogenic mutations were detected in MLH1 and PMS2 genes. However, methylation-specific PCR analysis confirmed the hypermethylation on MLH1 promoter. Additionally, no mutation was found in the EPCAM, KRAS, NRAS and BRAF genes.

**Table 1:** Pathogenic variations with clinical significance detected through next-generation sequencing (NGS).

Gene	Transcript	Alteration	Mutation type
MSH2	NM_000251.1	c.983delC (p.A328fs)	Frameshift
MSH6	NM_000179.3	c.3254dupC (p.T1085fs)	Frameshift
TP53	NM_000546.5	c.C817T (p.R273C); c.C586T (p.R196*)	Missense Nonsense
PBRM1	NM_001405607.1	c.835delA (p.I279fs)	Frameshift
B2M	NM_004048.4	c.37_38del (p.L13fs)	Frameshift
DNMT3A	NM_022552.5	c.176delC (p.P59fs)	Frameshift
KMT2D	NM_003482.4	c.7061delC (p.P2354fs)	Frameshift
SOX9	NM_000346.4	c.1433dupC (p.T478fs)	Frameshift
ERBB3	NM_001982.4	c.G310A (p.V104M)	Missense
PIK3R1	NM_181523.3	c.A1699G (p.K567E)	Missense
SETD2	NM_014159.7	c.913delA (p.T305fs)	Frameshift
KMT2B	NM_014727.3	c.644delC (p.T215fs); c.A3052T (p.K1018*)	Frameshift Nonsense
ERF	NM_006494.4	c.896delG (p.G299fs)	Frameshift
RNF43	NM_017763.6	c.C1111T (p.R371*)	Nonsense

Furthermore, the genomic profiling confirmed a high tumor mutational burden (TMB-H), 66.4 mutations/Mb, extremely higher than the reference value 6.6 mutations/Mb (**Figure 3C**). This result was consistent with the hypermutator phenotype caused by dMMR.

Critically, comprehensive germline testing revealed no pathogenic germline variants in the MMR genes or other cancer susceptibility genes. This finding effectively rules out Lynch syndrome as the underlying cause of the MMR deficiency in this case, suggesting a purely somatic origin for the molecular alterations.

## Discussion

This case presents a rare duodenal adenocarcinoma exhibiting a complete loss of all four MMR proteins. The predominant pattern of MMR deficiency in gastrointestinal cancers involves the concurrent loss of specific protein pairs due to their functional dimerization: MLH1 with PMS2, or MSH2 with MSH6. Isolated loss of PMS2 or MSH6 is also well-documented. However, the concomitant loss of all four MMR proteins is an exceptionally rare phenomenon in the literature. Reported cases are often attributed to the coincidental occurrence of two independent molecular events, such as somatic MLH1 promoter hypermethylation (causing loss of the MLH1/PMS2 pair) coupled with an incidental, potentially secondary mutation in the MSH2 or MSH6 gene (leading to loss of the MSH2/MSH6 pair) (14, 15). Fewer than 10 cases of complete MMR protein loss have been reported to date, primarily involving colorectal and small bowel cancers. The underlying mechanism mainly involves pathogenic variants in MSH genes (11, 12), and none have been reported in a molecularly validated duodenal adenocarcinoma featuring comprehensive molecular profiling.

Our case is particularly noteworthy because the IHC findings were corroborated by NGS, which identified somatic mutations in both MSH2 and MSH6, and suggested an epigenetic mechanism of MLH1 promoter hypermethylation for the loss of the MLH1/PMS2 pair. The pan-MMR deficiency in this case was driven by a “double-hit” mechanism, characterized by the combination of genetic and epigenetic disruptions. The exceptional rarity of such a complete mechanistic presentation makes this case a valuable contribution to the literature.

The MMR deficiency leads to a failure to correct errors during DNA replication, resulting in a hypermutated phenotype and MSI-H (2, 3, 16). This will contribute to a high tumor mutational burden, as was confirmed in this case. The accumulation of numerous mutations can generate neoantigens that make these tumors highly visible to the host immune system (17). While this often correlates with a prominent tumor-infiltrating lymphocyte response and the exceptional efficacy of immune checkpoint inhibitors (ICIs) in this tumor subgroup; further more indicates a more favorable prognosis (17, 18). Tumors with dMMR/MSI-H have demonstrated profound and durable responses to ICIs across multiple cancer types, leading to their FDA approval for tissue-agnostic use in advanced dMMR solid tumors (19, 20). This case represents the critical therapeutic biomarkers, indicating a high likelihood of benefit from immunotherapy, which is a pivotal consideration especially in the setting of advanced or metastatic disease where traditional chemotherapy offers limited efficacy. KRAS/NRAS wild-type status and absence of the BRAF V600E mutation suggest potential sensitivity to cetuximab and panitumumab in this case. Additionally, the NGS profiling revealed the presence of variations in several immunotherapy-related biomarkers, including three positive predictors (TP53 p.R273C; TP53 p.R196*; PBRM1 p.I279fs), two negative predictors (B2M p.L13fs; IFNGR1 p.S378fs), and one hyperprogression-associated factor (DNMT3A p.P59fs) (21, 22). Other genetic alterations (PIK3R1 p.K567E; KMT2D p.P2354fs; SOX9 p.T478fs; ERBB3 p.V104M; SETD2 p.T305fs; KMT2B p.T215fs; KMT2B p.K1018*; ERF p.G299fs; RNF43 p.R371*; RNF43 c.252 + 1G>A) associated with tumorigenesis or cancer progression were also detected (23, 24). The functional relevance of these gene variants in this particular context warrants additional investigation.

SBAs, particularly those arising in the duodenum, are uncommon neoplasms, and the prevalence of dMMR/MSI-H is lower than in colorectal cancer (20). The clinical behavior and prognosis of dMMR SBAs are not as definitively characterized as in CRC, partly due to their rarity. Some studies suggest that like in CRC, MSI-H may be associated with a better prognosis in early-stage SBA (25, 26), but this remains an area of active investigation. The true paradigm shift, however, lies in the management of advanced disease. For patients with dMMR SBA, ICIs have emerged as a transformative treatment option. Case reports and series, along with subgroup analyses of larger clinical trials, have consistently shown remarkable response rates to pembrolizumab or nivolumab in this population (19, 20). This offers a potent, often less toxic, alternative to conventional chemotherapy. The present case, with its unequivocal dMMR status, underscores the absolute necessity of routine MMR IHC or MSI testing in all patients with duodenal and small intestinal adenocarcinomas to identify those who are candidates for these potential therapies. The patient was treated with adjuvant immunotherapy (Toripalimab) for a duration of 6 months after surgery, based on the dMMR/MSI-H/TMB-H profile. The treatment was well-tolerated with no significant immune-related adverse events reported. The postoperative course has been uneventful. At the last follow-up visit (6 months post-surgery), radiographic assessment showed no evidence of local recurrence or distant metastasis. Further clinical follow-up on this patient’s treatment responses and outcomes will be essential.

## Conclusion

In summary, this case report describes a rare duodenal adenocarcinoma with distinct clinicpathology and molecular features, including: 1) a rare primary location (duodenum); 2) an exceptionally rare phenotype of complete loss of all four MMR proteins; 3) MSI-H and TMB-H status, driven by a “double-hit” mechanism involving concomitant somatic mutations in the MSH2 and MSH6 genes alongside MLH1 promoter hypermethylation.

## Data Availability

The original contributions presented in the study are included in the article/Supplementary Material. Further inquiries can be directed to the corresponding author.
